# Epidermolysis bullosa House Austria and Epidermolysis bullosa clinical network: Example of a centre of expertise implemented in a European reference network to face the burden of a rare disease

**DOI:** 10.1007/s00508-016-1133-3

**Published:** 2016-12-01

**Authors:** Martin Laimer, Gabriela Pohla-Gubo, Anja Diem, Christine Prodinger, Johann W Bauer, Helmut Hintner

**Affiliations:** Department of Dermatology, Paracelsus Medical University Salzburg, Muellner Hauptstraße 48, 5020 Salzburg, Austria

**Keywords:** Orphan diseases, European Union, Epidermolysis bullosa, CLINET, EB House

## Abstract

Accurately addressing the diverse and complex issues of rare diseases (RD) in terms of prevention, recognition, diagnosis, treatment, care and research along key RD specificities, such as great heterogeneity, a limited number of patients, scarcity of relevant knowledge and expertise as well as enormous costs for patient care is a challenging task for healthcare providers and authorities that makes a supranational approach particularly feasible. The European Union has acknowledged RD matters by several initiatives, including efforts to implement national centres of expertise and European reference networks as well as a cross-border referral mechanism to foster access to expert services and to boost dissemination of clinical expertise and research activities. Exemplified by the EB House Austria, a centre of expertise for epidermolysis bullosa cross-linked with international reference partner institutions, this strategy proves its potential to be translated into optimized patient care and to meet the major medical, scientific, social and health-economic impact of RD.

## Definition and impact of rare diseases

According to Regulation (EC) N. 141/2000 of the European Parliament and of the Council, rare or orphan diseases (RD) are defined by a prevalence of 1 out of 2000 or less [1]. The vast majority of RD (i. e. 80%) have a genetic origin, while (auto) immunological, oncological and toxicological causes are found in only 10%, 4% and 3%, respectively (plus 3% others) [2]. The disease burden by RD is often considerable. Patients suffer from a chronic and often debilitating, progressive disease course with significant morbidity or even mortality. Treatment is mostly symptomatic. With respect to diagnostics, clinical parameters alone can be misleading and their interpretation challenging because (prototypic) findings, if appearing at all can be transient, inconsistent or not obvious in early infancy, when an accurate diagnosis is most critical. These limitations are further aggravated by the lack of standardized clinical data due to the rarity of RD and also by a marked genotypic variability of mutation-based disorders. These features of RD account for the occurrence of misdiagnoses and non-diagnoses, resulting in additional impairment and inadequate care, despite the fact that some RD are compatible (also by the use of orphan drugs) with a normal life if diagnosed on time. Socioeconomically, RD may not only significantly compromise educational and professional opportunities as well as social life but also cause enormously high costs, because treatment and management strategies are often very expensive and life-long. Moreover, cost-intensive regular follow-up and thorough examinations of RD patients are usually necessary in those mostly multisystem disorders. The limited access to clinical expertise and experience in diagnostic, therapeutic, organizational, bureaucratic, financial and research terms is mainly due to the small, seemingly negligible size of the respective patient cohort in each country, a major burden that makes RD often “the poor cousin” of public health policy, pharmaceutical industry and research funding.

### The relativity of being rare – epidemiological impact of rare diseases

Despite their rarity, RD have a significant impact on public health and socioeconomic matters that particularly increases with a supranational perspective. Thus, a prevalence of 1:2000 or less equates epidemiologically to up to 250,000 individuals suffering from one distinctive RD at the European level, when taking into account that the EU currently consists of 28 member states with a population of more than 500 million. Further considering 5000–8000 currently identified RD, those entities cumulatively affect about 6–8% of the general population, thus representing a significant proportion of approximately 27–36 million affected Europeans resulting in an enormous public health burden [3]. Of these individuals with an obvious need for special care 75% are children or adolescents, highlighting the importance of preventive and prophylactic efforts to avoid or ameliorate disease complications if possible.

## Acknowledgment of RD by the European Union

The key specificities of RD, including great heterogeneity, limited number of patients, scarcity of relevant knowledge and expertise as well as enormous costs predispose them as a unique target for a very high added value of cooperative action at national and international levels. This has been repeatedly acknowledged by the EU, e. g. by the Orphan Medicinal Product Regulation to set up criteria for orphan designation in the EU and to describe the incentives (e. g. 10-year market exclusivity, protocol assistance and access to the centralized procedure for marketing authorization) to encourage research, development and marketing of medicines to treat, prevent or diagnose RD [1]. The issue of RD was furthermore addressed by the Commission Communication 697 in 2008 [3] and the subsequent Council Recommendation on an action in the field of rare diseases in 2009 [4]. The main objective of these activities is to improve the access and equity to efficient prevention, diagnosis, treatment, care and research for patients suffering from a RD throughout the European Union (EU). This includes an overall community strategy to support member states to integrate national actions and initiatives into comprehensive intersectoral national action plans/strategies as well as transnational reference networks for RD, thereby gathering national expertise on RD and pooling it together with European counterparts (Table 1). In Austria, the national action plan for RD was recently approved by the Federal Ministry of Health [5].

**Table 1:** Core indicators for RD national plans/strategies (adopted from [7])

**Background indicators (preparation of the action plan/strategy)**	
1.	Laws or equivalent official national decisions supporting the establishment and development of a rare disease (RD) action plan or strategy; approval of RD special status
2.	RD advisory committee
3.	Patient representation and empowerment in action plan development, monitoring and assessment
4.	Adoption of the EU definition of RD
**Content indicators**	
*Centres of expertise (CE)*	
5.	National policy to provide high-quality healthcare through establishment of CE on RD
6.	Number of CE adhering to the national policy
7.	Participation of CE in European reference networks
*Information*	
8.	National plans/strategies (NP/S) support to the development of/participation in a comprehensive national and/or regional RD information system
9.	Help lines for RD
*Knowledge, classification/coding, registries and research*	
10.	RD good clinical practice guideline development and implementation
11.	Standardized definition/classification and codification of RD by the healthcare system
12.	Standardized registries or data collection on RD
13.	RD research programs and/or projects
14.	Participation in cross-border European and international research initiatives on RD
*Therapies*	
15.	Number of orphan medical products (OMP) with a European Union marketing authorization and availability in the country
16.	Governmental system for compassionate use of medicinal products
*Social services*	
17.	Programs to support the integration of RD patients in their daily life
**Financial support indicators (implementation of the action plan/strategy)**	
18.	Policy to ensure long-term sustainability of the RD action plan/strategy
19.	Amount of public funds allocated to the RD action plan/strategy
20.	Specific public funds allocated for RD research
21.	Public funds specifically allocated for RD research projects per year since the plan started

An inventory of the initiatives, plans and strategies undertaken in the EU member states has been jointly produced by the EU Committee of Experts on Rare Diseases (EUCERD, www.eucerd.eu) and EUROPLAN (www.europlanproject.eu), aiming to promote the implementation of the EU recommendation on rare diseases in the EU member states [6]. Core indicators to monitor national plans or strategies on RD of the member states have been recently published [7] and are instrumental for the decision-making process related to the adoption, assessment and further development of public policies for RD (Table 1).

## Centres of expertise (CE) and European reference networks (ERN)

Implementation of centres of expertise (CE) at the national level and their supranational integration into European reference networks (ERN) at the EU level has been defined as a key element of a coordinated European RD strategy by the European Commission High Level Group on health services and medical care that brings together experts from all member states to work on practical aspects of collaboration between national health systems in the EU [8, 9].

The CE provide expert structures for the diagnosis, management and care of RD patients at highest standards in defined catchment areas (Table 2; [8]). They bring together and coordinate multidisciplinary skills to serve the medical, rehabilitation and palliative needs of RD patients with, as far as applicable, evidence of good outcomes (including national and European legal provisions, participation in internal and external quality schemes) and also in close collaboration with patient organizations and alliances, such as the European Organization for Rare Diseases (EURORDIS, www.eurordis.org) to bring in their perspective. Their high level of expertise and experience is documented, for instance, by the annual number of referrals and second opinions, and through peer-reviewed publications, grants, positions, teaching and training activities. The CE further provide specialized laboratories or other core facilities, demonstrate a strong contribution to research, connect with each other and patient groups, appropriate health and social care providers, relevant research groups and diagnostic laboratories at national and international level, educate and train healthcare professionals as well as elaborate and disseminate good practice guidelines and disease information.

**Table 2:** EUCERD recommendations on quality criteria for centres of expertise (CE) for rare diseases in member states (adopted from [8])

Good practice guidelines for diagnosis and care
Quality assurance and outcome measures
High level of expertise and experience
Appropriate capacity to manage RD patients and provide expert advice
Contribution to state of the art research
Capacity to participate in data collection for clinical research and public health purposes
Capacity to participate in clinical trials
Demonstration of a multidisciplinary approach (e. g. RD board)
Collaborations to assure the continuity of care between childhood, adolescence and adulthood as well as all stages of the disease
Links and collaboration with other CE at national, European and international level as well as patient organizations
Appropriate arrangements for referrals within individual member states and from/to other EU countries
Appropriate arrangements to improve the delivery of care and especially to shorten the time taken to reach a diagnosis
Consideration of E‑Health solutions (e. g. shared case management systems, expert systems for tele-expertise and shared repository of cases).

### EB House Austria as example of a centre of expertise

**Fig. 1:**
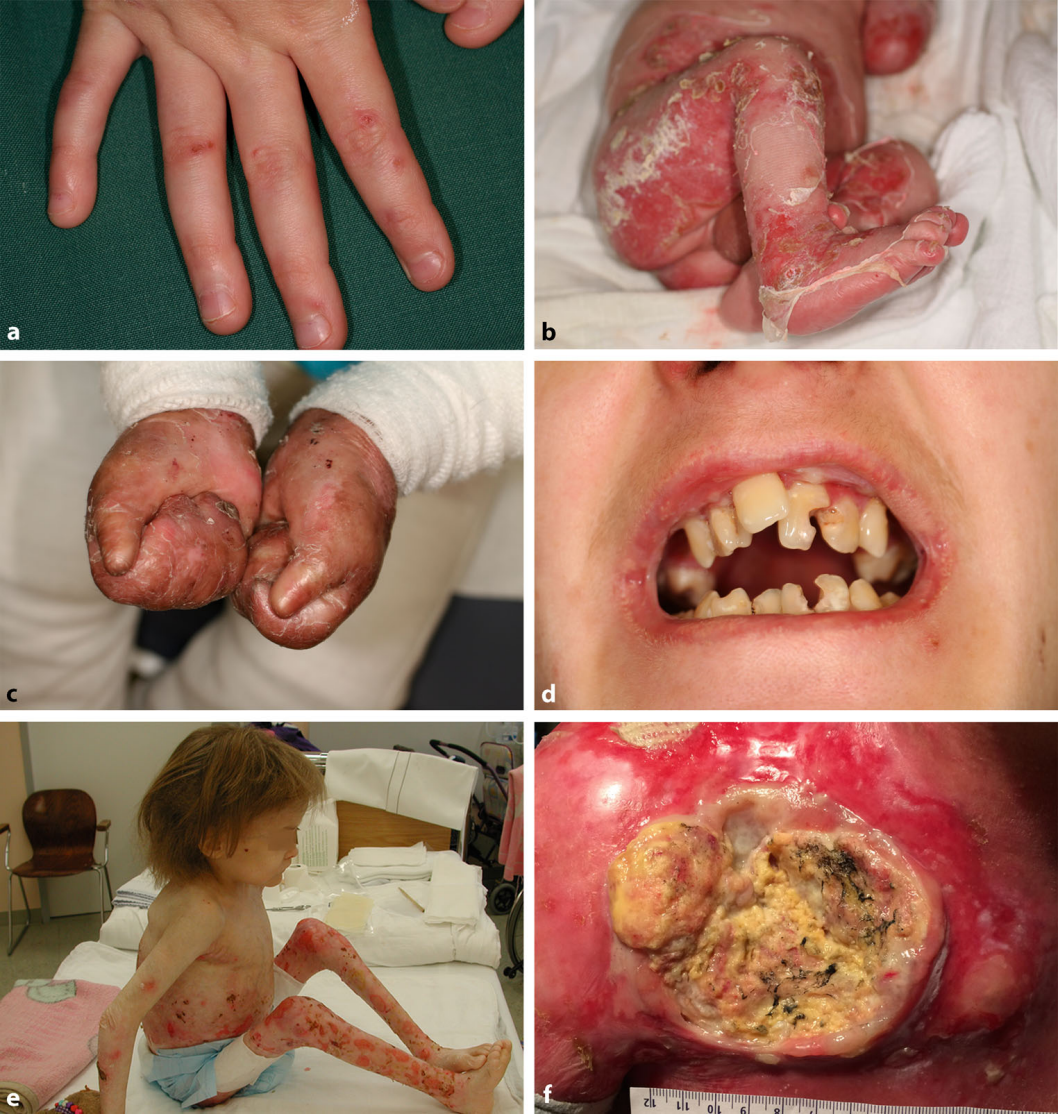
Phenotypic spectrum of epidermolysis bullosa(EB), a group of rare genodermatoses characterized by increased skin fragility as well as characteristic mechanically inducible blisters on the skin and mucous membranes due to mutational impairment of the structural and functional integrity of intraepidermal adhesion and dermoepidermal anchorage. As the mutated genes are also expressed in other epithelialized (e.g. gastrointestinal, respiratory and urogenital tracts) or mesenchymal (skeletal muscle) organs, extracutaneous manifestations and their complications render EB a multisystem disease associated with significant morbidity and mortality. **a** Localized variants, such as localized EB simplex present with (sometimes quite subtle) blistering and erosions, predominantly on mechanically exposed skin, such as the hands. **b** Generalized blisters, erosions, and large areas of denudation are the hallmark of the most severe subtype of generalized, severe junctional EB with early lethality due to sepsis, pneumonia or laryngotracheal obstruction by scarring and/or granulation tissue. **c** More chronic subtypes, such as generalized recessive dystrophic EB are complicated by excessive scarring especially at acral sites, leading to pseudosyndactyly (epidermal cocooning) with skin atrophy, nail loss, and contractures. **d** Intraoral disease with painful blisters and strictures predispose to microstomia, tooth deformities and enamel hypoplasia as well as excessive caries in generalized, intermediate EB. **e** Generalized severe recessive dystrophic EB with prominent (also extracutaneous) mucous membrane involvement commonly presents with complications, such as malnutrition, dystrophy, and growth retardation **f** Presumably based on chronically recurrent, inflammatory tissue traumatization and permanent reactive regeneration/hyperproliferation, squamous cell carcinomas occur disproportionately frequently and early (starting even in the second decade of life) especially in chronic wounds of patients with generalized severe recessive dystrophic EB. The course is highly aggressive with early metastatic spread

Dedicated to a multidisciplinary support of patients suffering from epidermolysis bullosa (EB, Fig. 1) and their families and devoted to the principles of best medical practice and state of the art care, the EB House Austria is one example for a CE (www.eb-haus.eu). It was founded in 2005 as an interdisciplinary clinical unit for diagnosis, medical care, academic affairs and research related to EB. The construction has been partly financed by public support but mainly by private donors and numerous initiatives, such as fund raising or publicity campaigns by the patient group DEBRA Austria (www.debra-austria.org). Designed as a national supply unit but also committed to the principles of equality, solidarity, and universality, the services of the EB House Austria are accessible to patients from all other countries worldwide and of course to those of all EU member states.

In the EB House Austria, expert consultation according to international standards, adherence to clinical practice guidelines (CPG), implementation of outcome measures, permanent quality control and independent validation (such as continuing evaluation of the EB registry) as well as demonstration of a multidisciplinary approach of care are basic principles. It operates through three distinct divisions:

- In the EB outpatient unit, specially trained and experienced medical personnel including 2 EB physicians, 2 EB nurses and further 15 EB experts of virtually all fields of medicine provide state of the art medical care, also including access to clinical studies on, e. g. orphan drugs/orphan medicinal research. Multidisciplinary management at one single location based on recommendations and decisions made at expert round tables (RD boards) and committed to principles of quality assurance is intended to allow early/timely and accurate, individualized care and best convenience according to principles for outpatients (all on 1 day/week) and surgeries (all in one). Patient services further include routine visits, visits on request and grand rounds; teledermatology as well as accessibility on call and emergency care day and night. A comprehensive review on the local EB experience was recently published in collaboration with international partners and is available in several languages [10].
- In the EB research laboratory, a research team works on (molecular) diagnostics, issues of basic research (e. g. carcinogenesis in EB) and advancement of techniques with the ultimate goal to develop a curative (molecular) therapy for EB. A close collaboration with the Salzburg University of Natural Sciences fosters transfer of biotechnological expertise, realization and accessibility of infrastructural core units as well as competitive consortial funding, e. g. granted by the EB patient organization DEBRA International on recommendations made by the DEBRA International medical and scientific advisory panel (MSAP), a committee of senior EB researchers and clinicians who, jointly reflect the breadth of EB research and oversee DEBRA’s centralized peer-review process of all research grant applications, thereby advising on research grant progress.
- Finally, the EB Academy provides continuous multidisciplinary education and training for lay persons, clinical professionals and scientists as well as administrates the EB registry Austria, which includes master data of currently more than 460 (thereof about 150 genetically characterized) EB patients. The registry data allow a better correlation of complex genotype/phenotype relationships, to determine epidemiological and prognostic markers, to identify and comprehensively characterize disease causing genes as well as pathogenic mutations and molecular pathways. It is further the basis of accurate prenatal and preimplantation diagnosis, carrier testing, classification as well as genetic counselling covering predictive diagnostics, prognostication and determination of recurrence risks. This is of considerable relevance, as despite recent progress no effective causative treatment for EB is currently available and prevention is still the main option available for couples at reproductive risk. The Academy further operates matters of public relations through print and electronic media (www.eb-haus.eu), organizes courses, congresses and in-coming/out-going expert visits as well as coordinates the EB-CLINET project (see later).

In Austria, the designation process of CE is currently under formal implementation (for detailed information refer to www.goeg.at). Approval of CE such as the EB House Austria is a consensual decision by all federal health care authorities to ensure a structured approach avoiding any kind of multiplication of efforts to boost the creation of a national network of equivalent institutions each specialized in one or more RD, with the goal of clinical, scientific and administrative collaboration. Including multisystemic diseases not restricted to one organ system, such management strategies for RD make a multidisciplinary approach mandatory to increase efficiency. Thereby, clinical management strategies and research efforts are focused and existing medical and genetic resources, infrastructure and collaborative networks are exploited.

## Together for more – European reference networks

At the continental level European reference networks (ERN) should provide a framework for healthcare pathways for patients with RD at a high level of integrated expertise, e.g. EUCERD recommendations on rare disease European reference networks (RD ERNS) [9]. Within ERN, CE are the core nodes. The network structures comprise a horizontal (CE at the same level, such as expert centres across different member states), vertical (different points of care of a healthcare pathway from primary care through to the CE providing expertise) and diagonal dimension (when different specialities such as social, rehabilitative and psychological specialists, physiotherapists and other services work together). The EU public health program 2020/2025 gives priority to RD ERN aiming at the introduction of 20–30 RD ERN until then.

On the background of the cross-border healthcare directive, which commonly along a national preauthorization procedure, allows accessibility of CE to all patients with conditions requiring a particular concentration of expertise or resources in medical disciplines that is not available in their home member state [11]. Key strategic elements of ERN include a defined governance, oversight structures, clear and comparable methods for evaluation and strong leadership qualities of co-ordinating site(s) to provide optimal cross-border services to all EU citizens (Table 3; [9, 12, 13]). The ERN should thereby provide access to the highest level of expertise, facilitate collaborative disease management and data collection for clinical research to, e. g. cope with recruitment issues, maximize the cost-effective use of European resources by concentrating them where appropriate and exploit innovations in medical science as well as health technologies (ERARE project for the improvement of RD research infrastructures; www.erare.eu).

**Table 3:** Objectives and major tools of European reference networks (ERN) to be accomplished (adopted from [9])

*Objectives*	Pooling of national expertise scattered throughout member states
	(Inter-) national collaboration and interoperability between CE, other ERN, national health and social care systems/providers, diagnostic and research laboratories, patients and individual experts within and between Member States
	Mobility and diffusion of expertise, information, knowledge, tools as well as quality and safety benchmarks (best practice) to facilitate treatment of patients in their proximity
	Reinforcement of research, epidemiological surveillance and training for health care professionals as well as developments of diagnosis and treatment of RD
*Major tools to accomplish these objectives*	Databases/registries/biobanks at disposal of the international research community with application of international terminologies to support interoperability
	Quality assurance and evaluation of performance (e. g. EuroGentest excellence network, www.eurogentest.org)
	Tools for tele-expertise (consultations, training, education) at disposal of the medical community
	Common guidelines/best standards of diagnosis, care, training and information
	European guidelines on diagnostic tests or population screening
	Certified medical training and pregraduate and postgraduate educational programmes in fields relevant to diagnosis and management of RD
	Communications infrastructure for visibility, accessibility and active recruitment of patients (Orphanet, national help lines, directories of expert services, pilot ERNs, EUCERD, information from patient organizations to assist health professionals);
	Sharing of Member States’ assessment reports on therapeutic or clinical value of orphan drugs at community level to minimise delays in access to orphan drugs for RD patients;
	Embedding of RD ERNs in the national and European healthcare systems and inter-network consortia with competitive cross-border funding to ensure sustainability

Relevant cost factors for ERN include project management, data collection and co-ordination in registries, information technology (IT), website and communication platforms, support for network meetings (within and between ERN), training and education packages (online, face to face), board activities as well as evaluation measures [9]. In this respect, the financial support from EU authorities is assumed to be proportional to the number of targeted patients, number of centres integrated as well as the number of diseases covered in terms of information to be produced and disseminated [9].

## EB-CLINET as example of a European reference network

EB-CLINET (www.eb-clinet.org) is a clinical network of EB centres and experts with currently 68 European and international partners in 52 countries worldwide. By linking clinical expertise in EB, the purpose of EB-CLINET is to share supranational competence and knowledge, to strengthen clinical cooperation, and to provide a basis for clinical trials. As an international collaboration, it operates on:

- data collection, initiation and coordination of a global EB register,
- elaboration and publication of clinical practice guidelines for standardization of care,
- implementation and publication of directories for EB centres of expertise (CE), EB laboratories and biobanks,
- and initiatives for professionals training and education.

Ultimately, EB families, wherever they live, should be able to receive professional high-quality support involving fair and dignified access to care services without the need for exhausting travel, language barriers or financial obstacles. The EB-CLINET has organized two international meetings in Salzburg, Austria, with over 90 delegates from 29 different countries around the world. The 4th EB-CLINET conference will take place in Salzburg in September 2017.

## Perspectives of molecular research in RD – from the rarest to the most common

The exponential advances in molecular biology made over the last two decades have elucidated the etiology of many of the RD. As a consequence, research on these diseases has ultimately proved to be very useful for a better understanding of the mechanism of very common conditions, as they may represent a model of dysfunction of a distinct biological pathway. These findings paved the way for a new era of targeted therapeutic interference, in dermatological terms to mention icatibant for hereditary and sporadic angioedema, vismodegib for basal cell carcinoma and Gorlin syndrome, as well as diacerein for EB [14–16].

Facing the challenging characteristics of RD and current limitations in care and support of patients with RD as outlined and likewise the promising perspectives (in research and therapy) and commitments by national and supranational healthcare authorities, the medical community should actively participate in the establishment and support of institutions of expertise for diagnosis, treatment and care of RD committed to the principles of reference in medical assistance, academics and research. In this spirit, the initiative of a reference network for genodermatoses (GERN) was launched at the 22nd Congress of the European Academy of Dermatology and Venereology (EADV) in 2013 in Istanbul. This networking activity was originally driven by the sole initiative of 25 participating experts from 13 countries and from 2 European and International patient networks and formally independent of the official application, assessment and approval procedures established by the European Commission for ERN that are currently ongoing.

Participants have proposed and discussed strategies to meet the three designated core components of GERN: disease registries, good clinical practice guidelines and best standards of diagnosis and care as well as training and education tools. Intended as a cluster of excellence, it should be built on pre-existing networks, such as the Fondation René Touraine (www.fondation-r-touraine.org), Geneskin (http://cordis.europa.eu/result/rcn/51882_en.html), EB-CLINET (www.eb-clinet.org) and the European Network for Ichthyosis e. V. (www.ichthyose.eu). The GERN is coordinated by the Fondation René Touraine Genodermatoses Network Committee in close collaboration with the EADV Genodermatoses Task Force Coordinators. GERN, currently involving 56 partners, also participated in the first call for applicants to provide a proposal for the establishment of (officially designated) ERN that was launched by the European Commission in Spring 2016 (http://ec.europa.eu/health/ern/policy/index_en.htm). The first ERN are expected to be formally approved and operational in Spring 2017.

## The win-win situation

Multidisciplinary care based on recommendations and decisions made at expert round tables and committed to the principles of quality assurance at CE will allow early and accurate care for patients in their proximity. Likewise, local healthcare providers will benefit from the reputation reflected by the efforts of such coordination centres for RD as well as from the cost-efficiency due to non-redundant but comprehensive and multidisciplinary diagnostics, focused assessment strategies and optimized treatment protocols. Concentrating and strengthening these efforts with respect to RD in collaborating reference networks will also facilitate governmental and private funding in times of progressively limited financial resources. Acquisition of research grants and international programs fostering research will stimulate the academic status of participating institutions. Overall, these intentions will have a major impact on scientific, medical, social and health-economic issues, raise public awareness, understanding and support and also improve the medical supply in the general population across Europe.

### Further information on rare diseases is available at the following links

- www.eurordis.org
- www.orpha.net
- www.europlanproject.eu
- www.eucerd.eu

If you are interested in genodermatoses, your expertise is very much appreciated! Please contact us and register your activity at office@genodermatoses-network.org.
